# Regional and Cellular Distribution of Nuclear Phosphorylated Tau (AT100) in the Frontal Cortex and Cerebellum of Cetaceans

**DOI:** 10.3390/biology15110845

**Published:** 2026-05-28

**Authors:** Belén Alonso-Estanillo, Maicol Ochoa, Laura Gómez, Xabier Pin, Alfredo López, Fernando Vásquez

**Affiliations:** 1 Faculty of Biomedicine and Veterinary Hospital, Alfonso X El Sabio University, 28691 Madrid, Spain; lcabanas@uax.es (L.G.); fvazqfer@uax.es (F.V.); 2 Faculty of Economics, Business and Communication, Universidad Europea, 28670 Madrid, Spain; 3 Coordinadora para o Estudo dos Mamíferos Mariños (CEMMA), 15570 Galicia, Spain; xabierpinvazquez@gmail.com (X.P.); cemmaorganizacion@gmail.com (A.L.)

**Keywords:** cetaceans, tau protein, AT100, brain aging, nuclear translocation, oxidative stress, bayesian analysis, neuroprotection

## Abstract

This study investigates the nuclear distribution of phosphorylated tau (AT100) in the frontal cortex and cerebellum of 12 cetaceans stranded along the Galician coast (NW Spain). Using Bayesian beta regression, we identified a suggestive positive trend between aging and tau phosphorylation, with posterior probabilities of 85.7% in the cortex and 89.7% in the cerebellum. Although the 95% credible intervals include zero in both models, reflecting the limited sample size, the direction and magnitude of the age effect were stable across multiple prior specifications and outlier-exclusion sensitivity analyses, suggesting a consistent age-related pattern rather than a data artefact. We hypothesize that the translocation of tau to the nucleus may act as a “nuclear shield” against cumulative oxidative stress, a process potentially intensified by the intermittent hypoxia characteristic of diving in these mammals; this hypothesis awaits direct empirical validation. A strong inter-regional correlation (r=0.932; Spearman ρ=0.923) points to a potentially coordinated regulation of tau phosphorylation across brain regions. Furthermore, markedly elevated AT100 levels in a subadult with cerebral necrosis suggest its potential as a marker of acute neuronal distress. These findings provide a preliminary baseline for understanding neuroprotection in cetaceans, with tau phosphorylation as a candidate mechanism for preserving neuronal genomic integrity under extreme physiological conditions.

## Simple Summary

This exploratory study investigates the nuclear distribution of phosphorylated tau (AT100) in the frontal cortex and cerebellum of 12 stranded cetaceans from Galicia. Using Bayesian beta regression, we found a suggestive age-associated trend in nuclear AT100 immunoreactivity, with posterior probabilities of 85.7% and 89.7% for the cortex and cerebellum, respectively. Although the 95% credible intervals include zero, consistent with the small sample size, the direction of the effect is stable across multiple sensitivity analyses. A strong inter-regional correlation (r=0.932; Spearman ρ=0.923) suggests a potentially coordinated pattern of tau phosphorylation across brain regions, independent of sex. We hypothesize that nuclear tau translocation may serve as a “nuclear shield” protecting neuronal genomic integrity against oxidative stress, a process potentially relevant to the intermittent hypoxia inherent to diving behaviour. This hypothesis requires direct validation through future studies incorporating oxidative stress markers and comparative data from terrestrial species. Additionally, markedly elevated AT100 levels in a subadult with cerebral necrosis suggest that this protein may also serve as a sensitive marker of acute neuronal distress, highlighting its potential dual utility. This preliminary work is limited by a small sample size (n=12) and multispecies heterogeneity, but establishes a baseline for future research on neuroprotection and brain aging in marine mammals.

## 1. Introduction

Brain aging is a complex and multifaceted process involving structural and functional changes that affect cognition, memory, and behavior. In humans, age-related declines in neuroplasticity have been reported and are thought to contribute to cognitive changes associated with normal aging [1,2,3,4]. From a comparative perspective, cetaceans and humans exhibit notable similarities in brain organization, including high levels of encephalization, cortical complexity, and refined neuronal architecture [5,6,7,8]. These convergent traits are associated with complex social behavior and have been proposed to reflect shared principles of neural organization that may extend to aspects of brain development and aging [9,10,11]. In this context, cetaceans constitute valuable models for comparative studies, particularly given the exceptional longevity observed in some species [12].

At the cellular level, tau protein regulates cytoskeletal stability through its interaction with tubulin [13]. Beyond its structural role, tau is implicated in cellular stress responses and age-related neurodegenerative conditions, including Alzheimer’s disease [14,15]. Experimental studies have demonstrated that tau can bind DNA and exert protective effects against oxidative stress [16,17,18,19]. Under physiological conditions, tau phosphorylation is a dynamic process that modulates cytoskeletal organization [20,21]. Among phosphorylated tau species, the AT100 epitope (Thr212/Ser214) is recognized by specific conformational changes [22,23]. In mammalian brains, nuclear localization of AT100 has been associated with neuronal aging and DNA protection [20,24,25]. Conversely, in pathological states, nuclear AT100 immunoreactivity decreases as cytoplasmic aggregation increases [15,20,26]. This duality highlights its relevance as a marker of neuronal state [27]. The present study aimed to evaluate the nuclear localization of AT100 in the frontal cortex and cerebellum of several cetacean species stranded along the Galician coast.

## 2. Materials and Methods

### 2.1. Study Subjects

A total of 12 stranded cetaceans from the Galician coast were analyzed between February and August 2025, representing four species: *Delphinus delphis* (n=8), *Phocoena phocoena* (n=2), *Tursiops truncatus* (n=1), and *Stenella coeruleoalba* (n=1). All carcasses were assigned a decomposition code of 1 (fresh) at necropsy, minimizing potential post-mortem autolytic effects on immunohistochemical results; however, systematic recording of exact post-mortem intervals was not performed, and this represents an acknowledged limitation of the dataset.

Samples were obtained during official necropsies of stranded carcasses conducted following standardized international protocols for cetacean necropsy by the authorized organization Coordinadora para o Estudo dos Mamíferos Mariños (CEMMA). The collection and handling of these biological materials were conducted in strict accordance with Spanish Law 42/2007 on Natural Heritage and Biodiversity, and Royal Decree 139/2011, which regulates the List of Wild Species under Special Protection and the Spanish Catalogue of Endangered Species. Furthermore, all procedures aligned with the European Marine Strategy Framework Directive (2008/56/EC) regarding the monitoring and protection of marine mammals. The study protocol was formally reviewed and approved by the Ethics Committee for Animal Experimentation of the Universidad Alfonso X El Sabio (UAX), Madrid, Spain (File No. 2025_02/353). Individuals were classified by sex and age category following the CEMMA expert criteria, which are based on total body length, external development, and dental eruption patterns, applied according to species-specific growth curves and sexual maturity markers [28,29]. When available, dental age estimation was performed by counting Growth Layer Groups (GLGs) [30,31,32]. Individuals were grouped into three classes: juveniles (minimal dental wear, incomplete sexual development), subadults (moderate wear, near-maximum body length), and adults (marked dental wear, complete sexual maturity). For statistical purposes, age was treated as an ordinal variable (1 = juvenile, 2 = subadult, 3 = adult), reflecting the directional biological progression of neural maturation and senescence. This ordinal treatment provides a parsimonious model that captures the trend of tau accumulation while maintaining statistical stability, particularly given that exact chronological ages were not uniformly available for all individuals. Likewise, as detailed in Table 1, frontal cortex and cerebellum samples were available for all specimens, including external observations regarding signs of incidental capture and net marks.

### 2.2. Sample Analysis

All biological samples were immediately fixed after collection in 4% (*v*/*v*) formaldehyde solution to ensure proper preservation of tissue architecture. Subsequently, tissues were processed using conventional histological techniques, including dehydration through ascending ethanol series and embedding in paraffin blocks. Serial sections were then cut from these blocks using a microtome for histological and molecular analyses, specifically following standardized and validated immunohistochemistry protocols established in this study.

For immunohistochemical studies, sections were deparaffinized in xylene and rehydrated through graded ethanol series. Antigen retrieval was performed by heat-induced epitope retrieval in citrate buffer (pH 6.0) at 95 °C for 20 min. Endogenous peroxidase activity was subsequently blocked with 3% hydrogen peroxide in methanol for 10 min, followed by blocking of nonspecific binding sites with 5% normal goat serum in PBS for 1 h at room temperature.

Sections were incubated with the primary antibody AT100 (Invitrogen (Thermo Fisher Scientific, Waltham, MA, USA), Phospho-Tau (Thr212, Ser214) Monoclonal Antibody (clone AT100, cat. MN1060)) at a 1:100 dilution in PBS containing 0.2% Triton X-100 for 2 h at room temperature. This dilution was selected following a preliminary titration series (range 1:50–1:200) to ensure maximal signal specificity and minimal background in cetacean nervous tissue. To support cross-reactivity in the study species, a bioinformatic sequence alignment (BLASTp) confirmed 100% amino acid identity at the AT100 epitope region (Thr212/Ser214) between humans and all four cetacean species analyzed (*D. delphis*, *P. phocoena*, *T. truncatus*, *S. coeruleoalba*), consistent with the high conservation of this proline-rich region across mammalian lineages [20]. Antibody binding was visualized using 0.025% 3,3′-diaminobenzidine (DAB Peroxidase Substrate Kit; Vector Laboratories, Newark, CA, USA) with 0.01% H_2_O_2_ as the catalytic agent. All sections from the 12 individuals were processed in the same staining batch to minimize inter-preparation variability. Finally, sections were mounted with Entellan (Merck KGaA, Darmstadt, Germany).

Neuronal nuclei in the frontal cortex and cerebellum were quantified by two trained pathologists from the Pathology Service of the Veterinary Clinical Hospital of Universidad Alfonso X El Sabio (HCV-UAX), who were blinded to the age and sex of the individuals, using a Leica DM 750 light microscope (Leica Microsystems, Wetzlar, Germany) equipped with a Leica digital camera (software version 1.0.1.8). Neurons were identified based on established morphological criteria, large, round to oval nuclei (diameter >8 μm), prominent nucleoli, and abundant cytoplasm, as well as their characteristic neuroanatomical location. Non-neuronal cells, including lymphocytes (small, dense, irregular nuclei), endothelial cells, and glial cells, were systematically excluded from the count. In cerebellar preparations, granule cells were distinguished from lymphocyte-like infiltrates by their small spherical nuclei, regular chromatin pattern, and specific organization within the *stratum granulosum*; any small mononuclear cells located in perivascular spaces or showing morphology inconsistent with local neuronal architecture were strictly excluded from the quantitative analysis. Five fields per immunohistochemical preparation were analyzed using three samples per animal at 600× magnification in sections of the frontal cortex and lateral cerebellar hemispheres. Inter-observer reliability was assessed using the intraclass correlation coefficient (ICC(A,1); two-way mixed model, absolute agreement, single measures) [33]. ICC values were 0.997 (95% CI: 0.990–1.000) for the cerebral cortex and 0.991 (95% CI: 0.970–1.000) for the cerebellum, indicating excellent agreement between observers. Mean counting bias was negligible in both regions (cortex: +0.76%; cerebellum: +0.62%), with limits of agreement of [−3.13%, +4.64%] and [−5.99%, +7.23%], respectively. Negative controls (sections processed without primary antibody) were not included in this exploratory study owing to limited tissue availability; this is acknowledged as a limitation and should be addressed in future validation studies.

### 2.3. Statistical Analysis

Due to the small sample size (n=12) and the proportional nature of AT100 expression data (percent values ranging from 0 to 100%), Bayesian statistical methods were employed. These are particularly suitable for limited datasets as they allow the incorporation of prior biological knowledge and provide full uncertainty quantification via credible intervals [34,35]. It should be noted, however, that with small samples, posterior estimates remain sensitive to prior specification; poorly chosen priors can introduce bias that may exceed that of frequentist alternatives. To address this concern, informative priors were grounded in published mammalian tau literature, and two complementary sensitivity analyses were conducted: one assessing robustness to prior specification (Section 3.6), and one assessing robustness to the influence of atypical individuals (Section 3.7).

#### 2.3.1. Bayesian Beta Regression Models

AT100 expression data were modeled using Bayesian Beta regression, appropriate for proportional data bounded between 0 and 1 [36]. The Beta distribution naturally accommodates the constraints of percentage data and the various distributional shapes commonly observed in biological proportions. Separate models were fitted for AT100 expression in the cerebral cortex and cerebellum:

(1) $${\mathrm{AT}100}_{i}∼\mathrm{Beta}\left(\mu_{i},\;\kappa \right)$$

(2) $$\mathrm{logit}\left(\mu_{i}\right)=\alpha +\beta_{\mathrm{age}}\cdot {\mathrm{age}}_{i}+\beta_{\mathrm{sex}}\cdot {\mathrm{Sex}}_{i}$$

where μ represents the expected proportion of AT100-positive neurons, κ is the concentration parameter (inversely related to variance), Age is an ordinal variable (1= juvenile, 2= subadult, 3= adult), and Sex is a binary indicator (1= male, 0= female).

The ordinal coding of age assumes approximate equidistance between consecutive classes; while this assumption is a biological simplification, it is consistent with the three-class developmental framework commonly applied in cetacean studies [28,29] and is supported by the exploratory nature of this pilot analysis.

#### 2.3.2. Prior Specification

Informative priors were defined based on existing literature on tau protein expression in mammalian brains [15,37,38,39,40]. For cortex models, the prior for the intercept was set as N(logit(0.6),0.5), reflecting expected baseline AT100 levels of ∼60% in young cetaceans. For cerebellum models, a slightly lower baseline was assumed: N(logit(0.5),0.5). Age effects received weakly informative positive priors: N(0.1,0.2), based on literature suggesting increased tau phosphorylation with age. Sex effects were assigned noninformative priors centered at zero: N(0,0.2). The concentration parameter κ was assigned an Exponential(0.5) prior, favoring moderate dispersion while allowing high variability if supported by the data.

#### 2.3.3. Model Fitting and Diagnostics

Models were fitted using PyMC (version 5.0+; PyMC Developers; available at https://www.pymc.io) with No-U-Turn Sampling (NUTS), a Hamiltonian Monte Carlo variant effective for complex hierarchical models [41]. Four chains were run with 2000 warm-up and 2000 posterior samples each, yielding a total of 8000 posterior samples. Convergence was assessed via the Gelman–Rubin statistic ($\hat{R}<1.01$) and effective sample sizes (ESS>400). Model fit was evaluated through posterior predictive checks, comparing observed data distributions with distributions predicted from posterior parameter samples.

#### 2.3.4. Species Covariate Assessment via LOO-CV

To formally assess whether species identity should be included as a covariate, the base model was compared against an extended model including a binary species indicator (*D. delphis* versus other species) using leave-one-out cross-validation (LOO-CV) based on the expected log pointwise predictive density (ELPD) [42]. A negative ΔELPD (base minus extended) would indicate improved predictive performance with species included; a positive value indicates no benefit from the additional covariate.

#### 2.3.5. Interpretation Framework

Bayesian results were interpreted probabilistically rather than via classical null hypothesis testing. Posterior means with 95% credible intervals (CrI) are reported. The probability of positive effects, P(β>0), provides a direct measure of the evidence for age-related increases. Critically, when the 95% CrI includes zero, results should be interpreted as *suggestive* of a trend rather than conclusive evidence, as the data remain statistically compatible with a null age effect. Effect sizes are reported on the logit scale and also back-transformed to percentage-point (pp) differences between juveniles and adults to facilitate biological interpretation.

### 2.4. Declaration of Generative AI in the Writing Process

During the preparation of this manuscript, the authors used Gemini 3.1 Flash-Lite (Google LLC, Mountain View, CA, USA) for language editing and proofreading, and Claude Sonnet 4.6 (Anthropic PBC, San Francisco, CA, USA) to assist code debugging and manuscript revision. No Generative AI was used for data collection, experimental design, or interpretation of results. The authors reviewed all AI-assisted content and take full responsibility for the final version.

## 3. Results

### 3.1. Immunohistochemical Analysis and AT100 Quantification

Manual quantitative evaluation of AT100-positive nuclei is summarized in Table 2.

**Figure 1:**
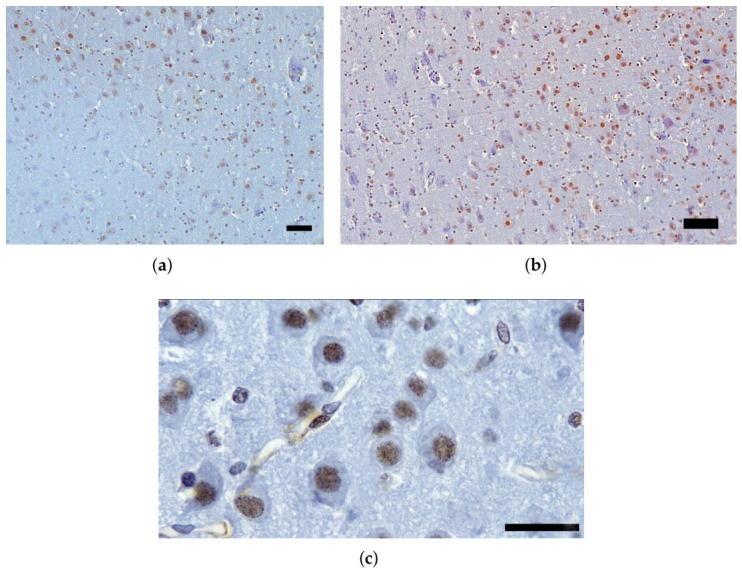
AT100 immunolabeling in the frontal cortex (*D. delphis*). Brown staining (DAB) in the neuronal nucleus indicates the nuclear localization of phosphorylated tau. (**a**) Juvenile individual showing low nuclear immunoreactivity (400×). (**b**) Adult individual displaying abundant and intense nuclear staining (400×). (**c**) Representative high-magnification detail (600×) confirming the strict localization of AT100 within the nuclear compartment at the single-cell level. Scale bars: (**a**–**c**) = 100 µm.

**Figure 2:**
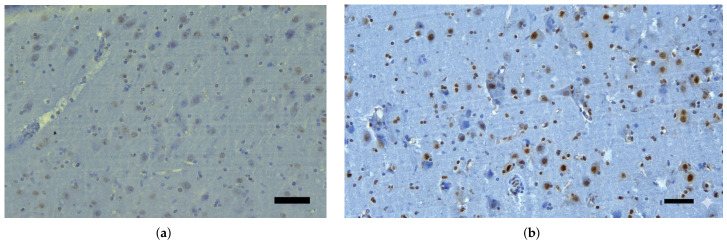
AT100 immunolabeling in the frontal cortex (400×) (*P. phocoena*). Brown staining (DAB) indicates AT100 immunoreactivity in the neuronal nucleus. (**a**) Juvenile individual showing low nuclear immunoreactivity. (**b**) Subadult individual displaying abundant and intense nuclear staining. Scale bars: (**a**,**b**) = 100 µm.

The immunohistochemical analysis of AT100 protein generally revealed age-related differences in its subcellular distribution in both frontal cortex and cerebellum. High-magnification analysis (600×) confirmed that AT100 immunoreactivity was strictly confined to the nuclear compartment at the single-cell level (Figure 1c). In the cortex, quantification of positive nuclei showed a higher proportion of nuclear AT100 immunoreactivity in adult individuals, followed by subadults, whereas juveniles exhibited lower nuclear immunoreactivity and a more restricted distribution (Figure 1 and Figure 2).

**Figure 3:**
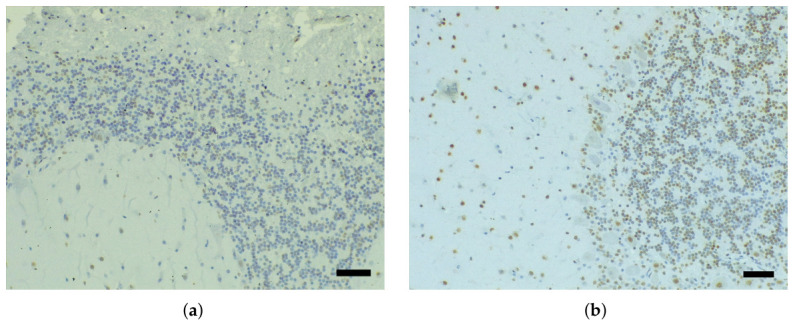
AT100 immunolabeling in the cerebellum (400×) (*D. delphis*). Brown staining (DAB) indicates AT100 immunoreactivity in the neuronal nucleus. (**a**) Juvenile: faint nuclear signal. (**b**) Adult: intense nuclear staining, mainly in granule cells. Scale bars: (**a**,**b**) = 100 µm.

**Figure 4:**
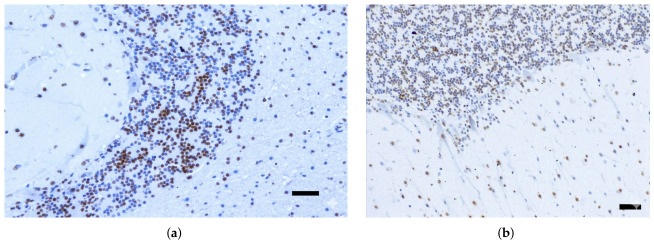
AT100 immunolabeling in the cerebellum (400×) (*P. phocoena*). Brown staining (DAB) indicates AT100 immunoreactivity in the neuronal nucleus. (**a**) Juvenile: faint nuclear signal. (**b**) Subadult: more extensive nuclear staining in granule cells. Scale bars: (**a**,**b**) = 100 µm.

As previously mentioned, the cerebellum exhibited the same age-dependent pattern, with adults showing the highest proportion of AT100-positive nuclei, followed by subadults, while juveniles displayed the lowest nuclear expression. Although quantification of AT100 in the cerebellum showed a progressive increase with age, values were generally lower than those recorded in the cortex for the same age groups. AT100 immunolabeling in the cerebellum revealed phosphorylated tau predominantly in granule cells and, to a lesser extent, in Purkinje cells (Figure 3 and Figure 4).

Overall, these results indicate a progressive increase in nuclear AT100 localization associated with advancing age in both analyzed brain regions.

### 3.2. Atypical AT100 Expression in Two Subadult Individuals

**Figure 5:**
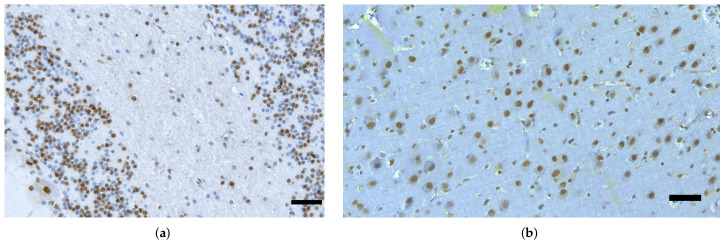
AT100 immunolabeling in the cerebellum (**a**) and cortex (**b**) (400×) of an atypical subadult male *D. delphis* presenting macroscopic cerebral necrosis at necropsy (AT100 CC: 96.55%; Cb: 62.41%). Brown staining (DAB) indicates AT100 immunoreactivity, visible primarily in granule cell nuclei. This individual is the primary necrosis case excluded in sensitivity analysis S2 (Section 3.7). Scale bars: (**a**,**b**) = 100 µm.

Two subadult male *D. delphis* exhibited nuclear AT100 immunoreactivity levels markedly above the subadult group mean: the first reached 96.55% in CC and 62.41% in Cb; the second reached 88.7% in CC and 78.4% in Cb (Table 2, Figure 5). These values overlap with the adult range and are substantially higher than the remaining subadults. The first individual (96.55% CC) presented macroscopic cerebral necrosis at necropsy. Necrotic processes trigger energy failure, ionic imbalance, and activation of stress-sensitive kinases (GSK-3β, CDK5), all of which promote tau hyperphosphorylation and nuclear redistribution independently of age [20,21]. The second individual (88.7% CC) showed no gross neuropathology, raising the possibility of subclinical injury or individual biological precocity. Notably, neither individual showed external signs of incidental capture or perimortem trauma during the CEMMA necropsies. The influence of these two individuals on the statistical estimates is formally assessed in Section 3.7.

### 3.3. Descriptive Statistics

AT100 expression was successfully quantified in all 12 cetacean individuals across both brain regions. In the cerebral cortex, AT100 expression ranged from 17.9% to 96.7% (mean ± SD: 57.3±28.4%), while in the cerebellum it ranged from 19.5% to 91.7% (mean ± SD: 49.0±23.8%). The sample included 3 juveniles, 5 subadults, and 4 adults, with 7 males and 5 females distributed across four cetacean species (*D. delphis* n=8; *P. phocoena* n=2; *T. truncatus*n=1; *S. coeruleoalba* n=1).

**Figure 6:**
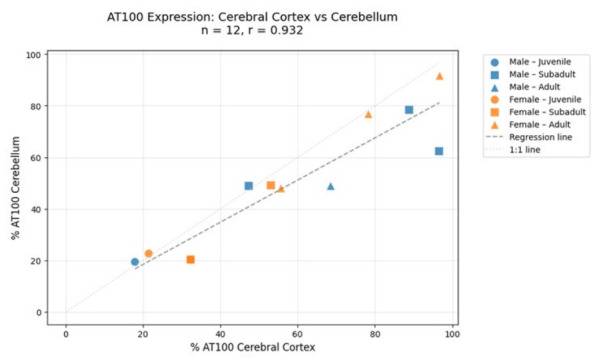
Correlation of AT100 expression between the cerebral cortex and cerebellum (n=12, Pearson r=0.932, Spearman ρ=0.923). Each point represents an individual, differentiated by age category (marker shape) and sex (color: blue = male, orange = female). The gray dashed line represents the regression line; the dotted line indicates the 1:1 relationship.

A strong positive correlation was observed between AT100 expression in the cerebral cortex and cerebellum (Pearson r=0.932; 95% CI: 0.74–0.98; Spearman ρ=0.923, p<0.001). The near-equivalence of the Pearson and Spearman coefficients indicates that this association is not an artefact of outlier leverage, and is maintained even when the two atypical subadults are excluded (Pearson r=0.961; Spearman ρ=0.902; n=10). These results suggest a potentially coordinated pattern of tau phosphorylation across brain regions within individuals. The distribution of individual values is illustrated in Figure 6.

### 3.4. Bayesian Regression Results

#### 3.4.1. Model Convergence and Fit

Both models showed excellent convergence diagnostics. All $\hat{R}$ values were ≤1.001, well below the threshold of 1.05, and effective sample sizes exceeded 4000 for all parameters. Posterior predictive checks confirmed adequate model fit in both brain regions.

#### 3.4.2. Age Effects on AT100 Expression

Bayesian analysis revealed a consistent positive age trend in both brain regions. In the cerebral cortex, the estimated age effect was ${\hat{\beta }}_{\mathrm{age}}=0.167$ (95% CrI: −0.139; +0.465), with an 85.7% posterior probability of a positive age effect. In the cerebellum, the age effect was slightly stronger: ${\hat{\beta }}_{\mathrm{age}}=0.199$ (95% CrI: −0.114; +0.495), with an 89.7% posterior probability of a positive effect.

Critically, the 95% credible intervals include zero in both models, indicating that the data are statistically compatible with a null age effect. These results should therefore be interpreted as suggestive of an age-related trend rather than as conclusive evidence. In a Bayesian framework, the posterior probabilities of 85.7% and 89.7% reflect the balance between the prior information and the observed data, and should not be treated as equivalent to a traditional hypothesis test. The consistently positive direction of the effect, stable across prior specifications and outlier-exclusion scenarios (see Section 3.6 and Section 3.7), provides meaningful preliminary support for an age-related pattern, while the uncertainty inherent to the small sample size (n=12) precludes stronger inferential claims. Back-transformation to the probability scale indicates that adults are predicted to show approximately +7.7 percentage points (pp) more AT100-positive neurons than juveniles in the cortex, and +9.7 pp more in the cerebellum, under the base model.

#### 3.4.3. Sex Effects on AT100 Expression

**Figure 7:**
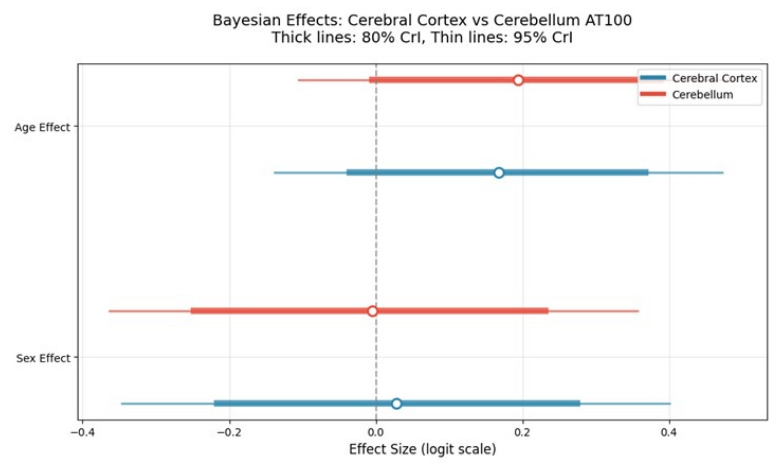
Bayesian effect estimates for the cerebral cortex (blue) and cerebellum (red). Thick lines: 80% credible interval; thin lines: 95% CrI; circles: posterior mean. Upper panel: age effect ($\beta_{\mathrm{age}}$), positive in both regions. Lower panel: sex effect ($\beta_{\mathrm{sex}}$), centered near zero, indicating no sexual dimorphism. All effects on the logit scale.

Sex effects were minimal and centered near zero in both brain regions. In the cerebral cortex, ${\hat{\beta }}_{\mathrm{sex}}=0.028$ (95% CrI: −0.346; +0.401), and in the cerebellum, ${\hat{\beta }}_{\mathrm{sex}}=-0.005$ (95% CrI: −0.364; +0.357). These results provide no evidence of sexual dimorphism in AT100 expression. Age and sex effects are shown comparatively in Figure 7.

### 3.5. Regional Comparison

**Figure 8:**
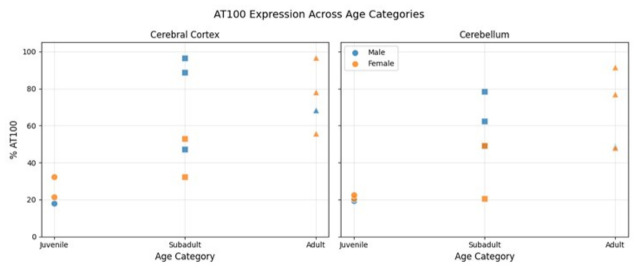
AT100 expression (% of positive nuclei) by age category in the cerebral cortex (**left**) and cerebellum (**right**). Circles: individual data points; males in blue, females in orange. A progressive increase with age is observed in both regions, consistent across sexes. Note the substantial inter-individual variability within each age category, particularly among subadults.

Comparison between the cerebral cortex and cerebellum revealed similar patterns of AT100 expression. Mean expression was slightly higher in the cerebral cortex (57.3%) than in the cerebellum (49.0%), yet age-related trends were nearly identical and the strong inter-regional correlation (r=0.932) suggests potentially coordinated tau phosphorylation mechanisms. Both regions displayed comparable age effect sizes and a similar absence of sex effects. The distribution of values by age category and sex in both regions is shown in Figure 8.

### 3.6. Sensitivity Analysis for Prior Specification

**Figure 9:**
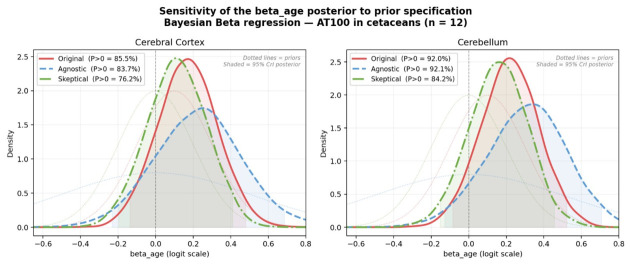
Sensitivity analysis for prior specification: posterior distributions of $\beta_{\mathrm{age}}$ under three prior specifications (Original, Agnostic, Skeptical) for the cerebral cortex (**left**) and cerebellum (**right**). Thin dashed lines indicate the reference priors; shaded bands represent the 95% CrI. The stability of the posteriors across scenarios indicates that the data support the direction of the effect independently of the prior used.

To assess the robustness of the Bayesian results to prior choice, a sensitivity analysis was conducted comparing three specifications for the prior of $\beta_{\mathrm{age}}$: the *Original* prior used in the main analysis [N(+0.10,0.20)], an *Agnostic* prior centered at zero with greater diffusivity [N(0.00,0.50)], and a *Skeptical* prior of equal precision to the original but with no directional bias [N(0.00,0.20)]. Figure 9 shows the posterior distributions of $\beta_{\mathrm{age}}$ under each scenario. All three posteriors exhibited substantial overlap and consistently pointed toward positive values, with $P(\beta_{\mathrm{age}}>0)$ ranging from 76.2% (Skeptical, cortex) to 92.1% (Agnostic, cerebellum), indicating that the direction of the age effect is supported by the data and is not an artifact of the informative prior.

**Figure 10:**
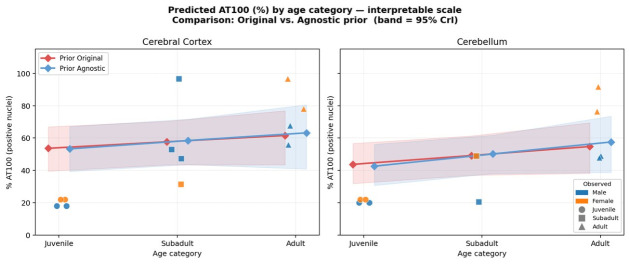
Predicted AT100 expression (%) by age category under the Original (red) and Agnostic (blue) priors, with 95% CrI bands. Observed data points overlaid (color = sex; shape = age category). Both priors agree on the direction of the age effect; the width of the bands reflects true uncertainty at n=12.

Note that the probabilities shown in Figure 9 and Figure 10 correspond to each specific prior scenario and therefore differ slightly from the base model estimates reported throughout the main text (cortex: 85.7%; cerebellum: 89.7%), which use the Original prior with the full n=12 dataset.

Figure 10 presents model predictions back-transformed to percentage points. Under the Agnostic prior, the predicted difference between a juvenile and an adult is +9.5 pp in the cortex and +13.9 pp in the cerebellum. The 95% credible interval bands are wide, reflecting the uncertainty inherent to the small sample size, but in all scenarios the predicted interval for adults lies above that of juveniles.

### 3.7. Sensitivity Analysis for Atypical Individuals

To formally assess whether the two subadults with markedly elevated AT100 expression exerted disproportionate leverage on the posterior estimates, two sensitivity analyses were conducted: (S1) excluding both atypical subadults (n=10); and (S2) excluding only the subadult individual with macroscopic cerebral necrosis (n=11).

**Figure 11:**
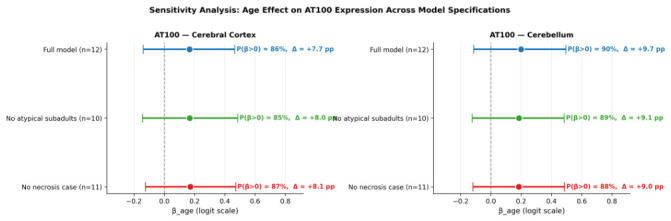
Forest plot of the age effect ($\beta_{\mathrm{age}}$) across three model specifications for the cerebral cortex (**left**) and cerebellum (**right**). Points indicate posterior means; error bars represent 95% credible intervals. The vertical dashed line at zero serves as reference. P(β>0) and the back-transformed juvenile-to-adult difference (Δ pp) are shown for each model. The near-identical estimates across both sensitivity scenarios confirm that the age-related trend is not driven by atypical individuals.

Results are summarized in Table 3 and illustrated in Figure 11. Across all specifications, the posterior probability of a positive age effect remained stable: $P(\beta_{\mathrm{age}}>0)$ ranged from 85.3% to 86.7% in the cerebral cortex and from 88.1% to 89.7% in the cerebellum. Posterior means and 95% credible intervals were virtually unchanged (cortex: ${\hat{\beta }}_{\mathrm{age}}=0.167$–0.172; cerebellum: ${\hat{\beta }}_{\mathrm{age}}=0.185$–0.199). These results confirm that the observed age-related trend is not driven by leverage from atypical individuals or pathological cases.

### 3.8. Species Covariate Assessment

To formally evaluate whether species identity should be included as a covariate, the base model was compared against an extended model including a binary species indicator (*D. delphis* vs. other) using LOO-CV. The base model showed superior predictive performance in both regions (cortex: ΔELPD=−0.047±0.142; cerebellum: ΔELPD=−0.323±0.121), providing formal justification for the exclusion of species from the primary analysis. In the cerebellum, the extended model was clearly penalized, with ΔELPD exceeding twice its standard error, indicating that the additional parameter introduces more uncertainty than it resolves given the available data.

## 4. Discussion

The present study provides preliminary evidence that adult cetaceans exhibit a higher proportion of neurons with nuclear immunoreactivity for phosphorylated tau (AT100) in both the frontal cortex and cerebellum compared with subadult and juvenile individuals. Our Bayesian analysis yields posterior probabilities of 85.7% and 89.7% for positive age effects in the cortex and cerebellum, respectively. While the 95% credible intervals include zero, reflecting the limited sample size, the direction and magnitude of the estimated effect are consistent across multiple prior specifications and outlier-exclusion scenarios, suggesting a genuine age-related pattern rather than a statistical artefact. These findings should be interpreted as hypothesis-generating and exploratory, and conclusions are appropriately limited to stranded coastal odontocetes from the NW Iberian coast, predominantly *D. delphis*.

The pattern of AT100 immunolabeling observed in the frontal cortex is consistent with findings reported in humans and other mammals [27,30]. Similarly, age-related increases in hyperphosphorylated tau and changes in its subcellular localization have been described in non-human primates such as *Callithrix jacchus* and in murine models [24,31,43]. The presence of comparable patterns across phylogenetically distant mammalian lineages suggests that tau phosphorylation may be linked to conserved biological processes. In this context, the AT100 epitope (Thr212/Ser214), associated with conformational changes in tau [20], may represent a candidate indicator of age-related neuronal modifications, though its role as a biomarker requires further validation.

The progressive increase in phosphorylated tau with age is likely driven by multiple interacting mechanisms, including imbalances between kinase activity (GSK-3, CDK5) and phosphatase function (PP2A), reduced efficiency of proteasomal and autophagic degradation, and increased oxidative stress [20,29]. Nuclear localization of tau has been proposed to play a protective role in maintaining DNA integrity and regulating chromatin organization under cellular stress [15,17,44], although this function may vary depending on the physiological or pathological context [21].

In the specific case of cetaceans, we hypothesize that this nuclear migration may be fundamentally linked to the extreme physiological demands of their environment. Unlike terrestrial mammals, cetaceans are frequently exposed to ischemia–reperfusion events during diving, which trigger significant production of reactive oxygen species (ROS). We propose, as a working hypothesis for future investigation, that nuclear p-tau translocation could represent a specialized response to an oxidative stress environment, acting as a “nuclear shield” to protect the genome against hypoxia-induced damage [18,19]. This hypothesis is conceptually supported by the diving physiology of cetaceans, but it was not directly tested in the present study; no direct measurements of oxidative stress markers, hypoxia indicators, or DNA damage were performed. Future studies incorporating such measurements and comparative data from terrestrial mammals are required to evaluate this hypothesis empirically.

It should also be noted that the functional role of nuclear tau remains an area of active debate. While protective functions, including DNA binding and chromatin stabilization, have been described [17,44,45], other evidence suggests that nuclear p-tau accumulation may also reflect early homeostatic disruptions, including interference with nucleocytoplasmic transport [46] or alterations in heterochromatin stability under pathological conditions [26]. The present data do not allow us to distinguish between protective and disruptive scenarios; this ambiguity reinforces the hypothesis-generating nature of our observations and underscores the need for mechanistic studies in cetacean tissue.

A particularly notable finding was the strong correlation between AT100 immunoreactivity in the frontal cortex and cerebellum (r=0.932; Spearman ρ=0.923). This association suggests that tau phosphorylation may be regulated through potentially coordinated, organism-level mechanisms rather than strictly region-specific processes. However, given the small sample size, this correlation estimate carries substantial uncertainty, and shared methodological or scaling factors cannot be fully excluded as contributing influences. The correlation should therefore be interpreted as consistent with coordinated tau regulation, while acknowledging that alternative explanations, including common responses to systemic factors such as stress hormones, body condition, or stranding-related physiology, remain plausible and warrant investigation.

In the cerebellum, phosphorylated tau was predominantly localized in granule cells and, to a lesser extent, in Purkinje neurons, a pattern consistent with previous observations in other mammals [28]. Granule cells play a central role in transmitting sensorimotor information and modulating Purkinje cell activity [34,35,36], and the observed distribution suggests that tau phosphorylation may influence cerebellar circuits involved in motor integration and coordination.

Two subadult male *D. delphis* exhibited nuclear AT100 immunoreactivity levels comparable to those of adults. The first (96.55% CC) presented macroscopic cerebral necrosis, with particularly elevated AT100 in both regions. Necrotic processes are associated with energy failure, ionic imbalance, and activation of stress-sensitive kinases, all of which can promote tau redistribution [21,29]. The second (88.7% CC) showed no gross neuropathology. Neither individual showed external signs of incidental capture, indicating that entanglement-related stress was not a contributing factor in either case. Importantly, the formal sensitivity analyses (Section 3.7) demonstrate that removing these individuals does not materially alter the posterior estimates, indicating that the age-related trend does not depend on their inclusion. However, the potential contribution of infectious agents cannot be excluded; pathogens such as morbillivirus, *Brucella ceti*, and *Toxoplasma gondii* are known to cause central nervous system lesions in marine mammals [47,48,49,50], and no formal infectious disease screening was performed in this study. Taken together, these cases suggest that elevated nuclear AT100 may reflect both physiological aging and acute neuropathological responses, supporting its potential dual utility as an aging marker and an indicator of neuronal distress.

A relevant observation that further supports the primacy of the aging effect over acute perimortem stress is the pattern observed in four young individuals (two juveniles and two subadults) in whom macroscopic inspection during CEMMA necropsies identified signs of incidental capture, such as net marks. Despite the acute perimortem hypoxia and stress typically associated with accidental entanglement, these individuals exhibited AT100 immunoreactivity levels substantially lower than those of the adult group, in which no evidence of capture-related trauma was present. This observation is important because it suggests that the acute physiological stress associated with incidental capture is not the primary driver of the progressive nuclear tau phosphorylation observed in our sample. Instead, the data are more consistent with the interpretation that physiological aging remains the predominant factor governing the increase and nuclear translocation of the AT100 epitope.

No significant differences were observed between males and females in AT100 immunolabeling patterns, consistent with the fundamental role of tau in neuronal homeostasis [20,21]. Our findings complement those reported by Orekhova et al. [37], who investigated β-amyloid and phosphorylated tau in Mediterranean cetaceans using the AT180 and AT8 epitopes. The AT100 epitope (pThr212/pSer214) differs from AT8 (pSer202/pThr205) and AT180 (pThr231) both in its phosphorylation sites and in the conformational requirements for antibody recognition [22]. These epitope-specific differences may explain the distinct distributions reported across studies, as each epitope may reflect a different aspect of tau pathophysiology, AT100 appearing more consistent with physiological aging processes in the present dataset, whereas AT8 and AT180 were more associated with pathological aggregates in the Italian cohort. Epitope-specific approaches are therefore important when comparing tau pathology across species and study contexts.

Regarding antibody specificity, the AT100 clone (Invitrogen, MN1060) is commercially validated for human and rodent tissue. Although formal cross-reactivity in cetacean tissue has not been independently demonstrated by Western blot or mass spectrometry, several converging lines of evidence support its applicability. A BLASTp alignment confirmed 100% amino acid identity at the AT100 epitope region (Thr212/Ser214) between humans and all four cetacean species analyzed, consistent with the high conservation of this proline-rich domain across mammalian lineages [20]. Critically, Orekhova et al. [37] successfully applied AT100 in *T. truncatus* and *S. coeruleoalba*, obtaining biologically coherent, age-related patterns consistent with ours. The consistent and age-dependent staining observed across all 12 individuals further supports antibody specificity in cetacean tissue. Nonetheless, formal validation via Western blot or peptide competition assays in cetacean brain tissue, as is common for non-model organisms, represents an important priority for future work.

The Bayesian analytical framework was appropriate for this study given the small sample size and the proportional nature of the outcome. The use of Beta regression ensured appropriate modelling of bounded data, and sensitivity analyses consistently supported the direction of the age effect. Nevertheless, several limitations must be explicitly acknowledged. First, the small sample size (n=12) constrains inferential power, and the 95% credible intervals encompassing zero should be taken seriously as evidence of substantial residual uncertainty. Second, the multispecies composition, while reflective of the opportunistic nature of stranding-based sampling, introduces biological heterogeneity that cannot be fully resolved at this sample size. LOO-CV formally confirmed that a binary species covariate did not improve predictive performance in either brain region, providing a data-driven rather than post hoc justification for the current analytical approach; however, adequately powered single-species studies remain a priority. Third, post-mortem intervals were not systematically recorded; although all specimens were assigned decomposition code 1 (fresh), factors such as the specific cause of death and potential perimortem hypoxic or inflammatory states could not be fully standardized. Variable pre-mortem stress, including stranding-related physiological changes, could theoretically influence the intensity or subcellular localization of phospho-tau; however, the fact that the age-related increase remained statistically robust across individuals and was not consistently present in cases of documented acute perimortem stress (e.g., incidental capture) suggests that the findings reflect a biological trend rather than a generalized post-mortem artefact. Fourth, negative immunohistochemical controls were not included owing to limited tissue availability; this constitutes a methodological gap to be addressed in future validation studies. Fifth, the scope of conclusions is limited to coastal odontocetes from the NW Iberian coast and should not be extrapolated broadly to all cetaceans without further evidence. Sixth, the representative immunohistochemical images were selected to illustrate the identified patterns and, while carefully chosen, are qualitative in nature. The quantitative strategy used, manual counting in a defined number of microscopic fields, validated by excellent inter-observer agreement (ICC > 0.99), lacks the systematic automation of digital image analysis and may not fully capture spatial heterogeneity of tau phosphorylation across entire brain regions. These sampling and quantification constraints should therefore be considered when interpreting the reported values as indicators of biological trends rather than absolute regional estimates.

## 5. Conclusions

This study provides the first description of the nuclear distribution of the AT100 phosphorylated tau epitope in the cetacean brain, and the first analysis of this protein in the cetacean cerebellum. Our results show a preliminary but consistent age-related trend in nuclear AT100 immunoreactivity in both the frontal cortex and cerebellum of stranded odontocetes from the NW Iberian coast, with a strong inter-regional correlation suggesting a potentially coordinated regulatory pattern. The direction and magnitude of the age effect are robust to multiple prior specifications and to the exclusion of atypical individuals, supporting the biological plausibility of the observed pattern despite the limitations of the pilot dataset.

We propose that nuclear tau translocation may represent an adaptive response to chronic oxidative stress and intermittent hypoxia in diving mammals, a “nuclear shield” hypothesis [17,44] that awaits direct empirical validation through measurements of oxidative stress markers and comparative studies in terrestrial mammals. Additionally, the observation of markedly elevated AT100 levels in an individual with cerebral necrosis suggests the potential dual utility of this protein as both an aging marker and a sensitive indicator of acute neuronal distress in cetaceans.

Despite the exploratory nature of this pilot study and the inherent limitations of the sample, the consistency of the patterns observed across species highlights the importance of investigating neuroprotective mechanisms in marine mammals. This work establishes a baseline for future research, and the analytical framework validated here, Bayesian beta regression with formal sensitivity testing, provides a replicable methodological template for forthcoming studies with larger and more taxonomically focused samples.

## Figures and Tables

**Table 1:** Distribution of cetacean specimens by species, sex, and age class. All individuals were assigned a decomposition code of 1 (fresh) at necropsy. Length and weight values are approximate.

Species	Age Class *	Sex	Length (cm)	Weight (kg)	External Observations
*Delphinus delphis*	Adult	M	218	95.2	No evidence of external trauma
*Delphinus delphis*	Adult	F	224	200.0	No evidence of external trauma
*Delphinus delphis*	Adult	M	194	110.3	No evidence of external trauma
*Delphinus delphis*	Juvenile	M	159	58.3	Signs of incidental capture (net marks)
*Delphinus delphis*	Subadult	M	196	75.6	Signs of incidental capture (net marks)
*Delphinus delphis*	** Subadult	M	196	74.8	No evidence of external trauma
*Delphinus delphis*	** Subadult	M	170	53.2	No evidence of external trauma
*Delphinus delphis*	Subadult	F	148	35.4	Signs of incidental capture (net marks)
*Tursiops truncatus*	Adult	F	278	223.0	No evidence of external trauma
*Stenella coeruleoalba*	Juvenile	F	126	23.1	No evidence of external trauma
*Phocoena phocoena*	Subadult	M	164	52.6	No evidence of external trauma
*Phocoena phocoena*	Juvenile	F	116	29.0	Signs of incidental capture (net marks)

* Age categories assigned according to body size, dental wear, and sexual maturity: juvenile, subadult, adult [28,29]. ** Subadult individuals showing adult-like AT100 expression; see Section 3.2. Bold formatting identifies the two atypical subadult individuals with AT100 expression markedly above the subadult group mean (>1.4 SD), as described in Section 3.2.

**Table 2:** AT100 expression data in cerebral cortex (CC) and cerebellum (Cb) for both observers. Counts expressed as total nuclei/AT100-positive nuclei (5 fields, 600×); AT100 (%) = percentage of positive nuclei. O1 = Observer 1; O2 = Observer 2.

Scientific Name	Age	Sex	CC O1		Cb O1		CC O2		Cb O2	
			Count	%	Count	%	Count	%	Count	%
*D. delphis*	Adult	M	156/122	78.2	156/120	76.9	154/120	77.9	154/118	76.6
*D. delphis*	Adult	F	140/96	68.5	159/78	49.0	142/98	69.0	161/80	49.7
*D. delphis*	Adult	M	140/78	55.7	156/75	48.0	138/75	54.4	155/74	47.7
*D. delphis*	Juvenile	M	140/25	17.9	154/30	19.5	141/28	19.9	152/32	21.1
*D. delphis*	Subadult	M	142/67	47.2	139/68	48.9	140/65	46.4	141/55	39.0
*D. delphis*	** Subadult	M	145/140	**96.55** *	149/93	**62.41** *	147/138	93.8	148/95	64.2
*D. delphis*	** Subadult	M	142/126	**88.7** *	156/123	**78.4** *	144/122	84.7	158/120	76.0
*D. delphis*	Subadult	F	147/31	32.2	158/32	20.5	145/50	34.5	156/35	22.4
*T. truncatus*	Adult	F	156/151	96.7	170/156	91.7	158/148	93.7	172/154	89.5
*S. coeruleoalba*	Juvenile	F	140/30	21.4	123/28	22.7	142/32	22.5	121/30	24.8
*P. phocoena*	Subadult	M	136/72	52.94	142/70	49.2	135/70	51.9	144/68	47.2
*P. phocoena*	Juvenile	F	147/47	32.2	156/32	20.5	148/45	30.4	154/34	22.1

* AT100 expression markedly above the subadult group mean; the individual with 96.55% CC presented macroscopic cerebral necrosis at necropsy (see Section 3.2). ** Both atypical subadults are male *D. delphis*. Bold values indicate AT100 expression markedly above the subadult group mean (see * footnote). ICC(A,1) between observers: 0.997 [0.990–1.000] (cortex); 0.991 [0.970–1.000] (cerebellum). Mean bias: cortex +0.76%; cerebellum +0.62%.

**Table 3:** Sensitivity analysis: posterior estimates of $\beta_{\mathrm{age}}$ across model specifications. Δpp = percentage-point difference between a predicted juvenile and a predicted adult (back-transformed from logit scale). CrI = 95% credible interval.

Model	*n*	Cerebral Cortex			Cerebellum	
		$\hat{\beta }$ [95% CrI]	P(β>0)	Δpp	$\hat{\beta }$ [95% CrI]	P(β>0)
Base (full)	12	+0.167 [−0.139; +0.465]	85.7%	+7.7	+0.199 [−0.114; +0.495]	89.7%
S1: no atypical SA	10	+0.168 [−0.144; +0.485]	85.3%	+8.0	+0.186 [−0.125; +0.484]	88.6%
S2: no necrosis case	11	+0.172 [−0.125; +0.472]	86.7%	+8.1	+0.185 [−0.119; +0.486]	88.1%

## Data Availability

The data presented in this study are available on request from the corresponding author.

## Abbreviations

The following abbreviations are used in this manuscript:

AT100	Phospho-Tau (Thr212/Ser214) monoclonal antibody clone AT100
CC	Cerebral cortex
Cb	Cerebellum
CEMMA	Coordinadora para o Estudo dos Mamíferos Mariños
CrI	Credible interval
DAB	3,3′-Diaminobenzidine
ELPD	Expected log pointwise predictive density
ESS	Effective sample size
GLG	Growth Layer Group
LOO-CV	Leave-one-out cross-validation
NUTS	No-U-Turn Sampler
pp	Percentage points
ROS	Reactive oxygen species
